# The ROS/GRK2/HIF-1*α*/NLRP3 Pathway Mediates Pyroptosis of Fibroblast-Like Synoviocytes and the Regulation of Monomer Derivatives of Paeoniflorin

**DOI:** 10.1155/2022/4566851

**Published:** 2022-01-29

**Authors:** Zhongyang Hong, Xianzheng Zhang, Tianjing Zhang, Ling Hu, Ruijin Liu, Pan Wang, Han Wang, Qianqian Yu, Dan Mei, Ziyang Xue, Feng Zhang, Lingling Zhang

**Affiliations:** ^1^Institute of Clinical Pharmacology, Anhui Medical University, Key Laboratory of Anti-Inflammatory and Immune Medicine, Ministry of Education, Anhui Collaborative Innovation Centre of Anti-Inflammatory and Immune Medicine, Center of Rheumatoid Arthritis of Anhui Medical University, Hefei 230032, China; ^2^Department of Pharmacy, Affiliated Fuyang Hospital of Anhui Medical University, Fuyang 236000, China

## Abstract

Hypoxia is an important factor in the development of synovitis in rheumatoid arthritis (RA). The previous study of the research group found that monomeric derivatives of paeoniflorin (MDP) can alleviate joint inflammation in adjuvant-induced arthritis (AA) rats by inhibiting macrophage pyroptosis. This study revealed increased levels of hypoxia-inducible factor- (HIF-) 1*α* and N-terminal p30 fragment of GSDMD (GSDMD-N) in fibroblast-like synoviocytes (FLS) of RA patients and AA rats, while MDP significantly inhibited their expression. Subsequently, FLS were exposed to a hypoxic environment or treated with cobalt ion in vitro. Western blot and immunofluorescence analysis showed increased expression of G protein-coupled receptor kinase 2 (GRK2), HIF-1*α*, nucleotide-binding oligomerization segment-like receptor family 3 (NLRP3), ASC, caspase-1, cleaved-caspase-1, and GSDMD-N. Electron microscopy revealed FLS pyroptosis after exposure in hypoxia. Next, corresponding shRNAs were transferred into FLS to knock down hypoxia-inducible factor- (HIF-) 1*α*, and in turn, NLRP3 and western blot results confirmed the same. The enhanced level of GSDMD was reversed under hypoxia by inhibiting NLRP3 expression. Knockdown and overexpression of GRK2 in FLS revealed GRK2 to be a positive regulator of HIF-1*α*. Levels of GRK2 and HIF-1*α* were inhibited by eliminating excess reactive oxygen species (ROS). Furthermore, MDP reduced FLS pyroptosis through targeted inhibition of GRK2 phosphorylation. According to these findings, hypoxia induces FLS pyroptosis through the ROS/GRK2/HIF-1*α*/NLRP3 pathway, while MDP regulates this pathway to reduce FLS pyroptosis.

## 1. Introduction

Rheumatoid arthritis (RA), a chronic aggressive and debilitating autoimmune disease with an unknown origin, is characterized by systemic inflammation response, the production of abnormal antibodies, and persistent synovitis [1, 2]. Approximately 9.7 million people worldwide are estimated to be suffering from this disease [3]. In recent years, hypoxia of synovial tissue has been increasingly recognized as an important factor influencing the development of RA [4–6]. Adaptive transcriptional responses to low oxygen tension are mediated mainly through the hypoxia-inducible factors (HIFs), which are tightly regulated by three prolyl hydroxylases and one asparagine hydroxylase [7]. In steady-state conditions, hydroxylases suppress the activity of HIF through the hydroxylation of proline (Pro402 and Pro564) of HIF-1*α*, leading to ubiquitination and proteasomal degradation. A further hydroxylation of Asn803 prevents HIF transcriptional activity [8]. These modifications allow HIF-1*α* to bind to Von Hippel-Lindau (VHL) for proteasomal degradation [9]. These processes are blocked by hypoxia, allowing HIF-1*α* to accumulate and induce the expression of HIF target genes [10].

Hypoxia induces excessive production of reactive oxygen species (ROS) through mitochondrial damage to increase the synthesis of HIF-1*α* [11, 12]. For example, the negative regulatory factor nonselenocysteine-containing phospholipid hydroperoxide glutathione peroxidase (NPGx) of the translational regulator cytoplasmic polyadenylation element-binding protein 2 (CPEB2) is consumed by ROS, leading to elevated HIF-1*α* RNA translation. A positive correlation between ROS and G protein-coupled receptor kinase 2 (GRK2) levels was found in sickle erythrocytes [13, 14]. Moreover, the phosphorylation of GRK2 serine 670 (S670) increased the total levels and cytoplasmic shuttling of the mRNA-binding protein human antigen R (HuR) in response to hypoxia, subsequently increasing the synthesis of HIF-1*α* in HeLa cells [15].

In a recent study, the expression of nucleotide-binding oligomerization segment-like receptor family 3 (NLRP3) was found to be mediated by HIF-1*α* during hypoxic conditions [16]. The integrated regulation of inflammasome activation and pyroptosis promotes the antimicrobial host defense and the clearance of pathogens [17, 18]. However, aberrant activation of NLRP3 inflammasome and pyroptosis may induce pathological autoimmune responses, that are harmful to the host [19–21]. For example, NLRP3 inflammasome is known to recruit and activate caspase-1 through an adaptor molecule ASC, and this activated caspase-1 can then cleave gasdermin D (GSDMD), interleukin (IL)-1*β*, and IL-18 to mature forms and subsequently trigger pyroptotic cell death [22]. Pyroptosis is redefined as GSDMD-mediated programmed necrosis, accompanied by the secretion of inflammatory cytokines IL-1*β* and IL-18 [23]. Both IL-1*β* and IL-18 belong to the IL-1 family and play vital roles in host defense, immune regulation, and inflammatory responses [24].

In RA, HIF-1*α* is highly expressed in synovial tissue, which contributes significantly to the expression of inflammatory genes and cell survival in the synovium [25]. Therefore, an effective RA treatment strategy to inhibit HIF-1*α* is urgently needed. In this regard, as the major active component of Paeonia lactiflora Pallas, Paeoniflorin (Pae) has been proven to prevent CoCl_2_-induced HIF-1*α* accumulation and the expressions of p53 and Bcl-2/adenovirus E1B 19 kDa interacting protein 3 (BNIP3) [26]. A preliminary study by our research group confirmed that Pae could inhibit inflammation in the animal models of autoimmune diseases, such as experimental arthritis, psoriasis in mice, and experimental autoimmune encephalomyelitis [26]. However, the bioavailability of Pae is not ideal due to its high hydrophilicity [27]. To this end, the monomer derivative of Pae (MDP), developed by our group, exhibited superior bioavailability and efficacy than Pae. MDP could reduce macrophage pyroptosis by inhibiting toll-like receptor 4 (TLR4) [28]. It is worth noting that arthritis is often accompanied by tissue hypoxia [29]. At present, there are only a few studies on the relationship between hypoxia of synovial tissue and pyroptosis, and MDP significantly reversed the pathological changes of synovial tissue in AA rats [28].

Thus, hypoxia in the microenvironment of the joint cavity exacerbates synovial inflammation in multiple ways. Fibroblast-like synoviocytes (FLS) pyroptosis may play a crucial role in the entire pathogenesis of RA. This programmed inflammatory cell death may be regulated by HIF-1*α*, and MDP may be an ideal drug to inhibit pyroptosis. In this study, we investigated the molecular mechanism of the correlation between hypoxia and FLS pyroptosis and the role of MDP in vitro. We hypothesize that elevated HIF-1*α* in FLS may aggravate synovitis via FLS pyroptosis. The increase in HIF-1*α* is partly due to the increase in GRK2 expression caused by oxidative stress. MDP may reduce the expression of HIF-1*α* through targeted inhibition of GRK2 and then inhibit FLS pyroptosis to alleviate synovial inflammation.

## 2. Materials and Methods

### 2.1. Antibiotics and Reagents

MDP [C_29_H_32_O_13_S, molecular weight: 620], purity > 98%, was provided by the Chemistry Laboratory of the Institute of Clinical Pharmacology of Anhui Medical University (Hefei, China); LW6 (CAS: 934593-90-5) was obtained from MedChemExpress (USA). N-acetyl-L-cysteine (NAC) was purchased from Beyotime Biotechnology (Shanghai, China). Dulbecco's modified Eagle's medium (DMEM) was obtained from Gibco Co. Ltd. (CA, USA). Fetal calf serum was purchased from Wisent Co. Ltd. (Canada). Streptomycin, penicillin, ROS Assay Kit, and goat anti-rabbit IgG were purchased from Beyotime Biotechnology Co. Ltd. (Shanghai, China). Enzyme-linked immunosorbent assay (ELISA) kits for IL-1*β* and IL-18 were the products from J&L Biological (Shanghai, China). Antibodies against GRK2, p-GRK2 S670, HIF-1*α*, NLRP3, ASC, Caspase-1, GSDMD, and *β*-actin were purchased from Affinity Bioscience (Taiwan, China). The antibody against VHL was the product from Wanleibio (Shenyang, China).

### 2.2. Isolation and Culture of Primary Fibroblast-Like Synoviocytes

This was performed as described earlier [30]. In brief, the synovial tissues of OA, RA patients, and healthy persons were washed 2-3 times with phosphate-buffered saline (PBS), then minced into 1-2 mm^3^ pieces. The tissue fragments were then transferred to the culture flask, placed in an incubator at 37°C and 5% CO_2_ for 4 h, then added into 3 mL DMEM supplemented with 20% fetal calf serum and antibiotics (100 U/mL penicillin, 100 *μ*g/mL streptomycin) for further culture until confluent (5-7 days) and then dissociated with Trypsin/EDTA into a single-cell suspension. After dissociation, fibroblasts were pelleted by centrifugation at 1,200 rpm for 5 min and cultured in DMEM supplemented with 20% fetal calf serum. FLS were identified based on both cell surface marker and morphological features as described previously [31]. The primary FLS of Sprague–Dawley rats were separated and cultured in the same way.

### 2.3. Hypoxia Treatment and Medication of Fibroblast-Like Synoviocytes

To observe the biological response of cells induced by hypoxia, FLS were exposed to hypoxia (1% O_2_ for 2 h or 24 h). Next, FLS were treated with or without MDP (10^−6^ mol/L), LW6 (a hypoxia-inducible factor 1 inhibitor; 5 *μ*mol/L), and NAC (100 *μ*mol/L).

### 2.4. Scanning Electron Microscopy (SEM) to Observe the Pyroptosis of Fibroblast-Like Synoviocytes

The cells were fixed with 5% glutaraldehyde, then dehydrated with gradient ethanol and hexamethyldisilazane, and placed in a fume hood overnight. The ion sputtering coating method was used for treatment of the cells. Images of the cells were acquired with an electron microscope (GeminiSEM 300, Carl Zeiss, Germany).

### 2.5. The Expression of Nucleotide-Binding Oligomerization Segment-Like Receptor Family 3, Hypoxia-Inducible Factor-1*α*, and Other Proteins in Fibroblast-Like Synoviocytes Was Detected by Western Blot

FLS were mixed with RIPA lysate (Beyotime Biotechnology) and ground for 10-15 min. Samples were agitated on ice for 30 min, and the supernatant was collected. The protein levels were quantified using a BCA protein assay kit (Roche, Basel, Switzerland). Then, the protein samples were resolved by sodium dodecyl sulfate-polyacrylamide gel electrophoresis to separate protein bands. Proteins were transferred from the gel onto polyvinylidene fluoride membrane and blocked with 5% nonfat dry milk for 2 h. The membrane was incubated overnight with the primary antibody (1: 500) at 4°C and then with the secondary antibody for 2 h. The bands were visualized by the electrochemiluminescence method, and the overall gray values of protein bands (average gray value area) were quantified. At the same time, *β*-actin was used as an internal marker to compare the gray value of target protein in different groups.

### 2.6. Analysis of N-Terminal p30 Fragment of GSDMD (GSDMD-N) Expression by Immunofluorescence

FLS were cultured in 35 mm glass-bottom dishes, fixed with 4% paraformaldehyde, permeabilized with 0.1% Triton X-100, and then, blocked with 0.5% bovine serum albumin (BSA). The cells were then incubated with anti-GSDMD-N (1 : 100) antibody in a 4°C wet chamber. Next, FLS were washed with PBS and then were incubated for 2 h at room temperature with Alexa-Fluor-594-tagged secondary antibodies. The images were detected by fluorescence microscopy (LEICA sp8), and positive images further underwent computerized digital image analysis. The intensity of immunofluorescence was analyzed using Image J.

### 2.7. Detection of IL-1*β* and IL-18 Levels by Enzyme-Linked Immunosorbent Assay

FLS were treated with or without MDP and then cultured in a 1% oxygen or normoxia environment for 24 h. The cell supernatant was collected for cytokine detection. In brief, IL-1*β* and IL-18 levels in the culture media were determined using commercially available rat IL-1*β* and IL-18 ELISA kits according to manufacturer's instructions.

### 2.8. Short Hairpin RNAs Were Used to Inhibit HIF-1*α* and NLRP3 Expressions in FLS

To study the role of HIF-1*α* and NLRP3 in the pathway of pyroptosis in FLS, the commercially available short hairpin RNAs for HIF-1*α*, NLRP3 (Table 1) and empty vector (General Biol, Chuzhou, China) were purchased. Then, FLS were transfected with shRNAs by using Lipofectamine 3000 (Invitrogen, CA, USA) according to manufacturer's instructions.

### 2.9. Short Hairpin RNA and Overexpression Plasmid to Alter G Protein-Coupled Receptor Kinase 2 Expression in FLS

To verify whether GRK2 can promote the expression of HIF-1*α*, the commercially available GRK2 (Table 1) and empty vector (General Biol, Chuzhou, China) were used. The commercially available GRK2 overexpression plasmid and empty vector were purchased from General Biol (Chuzhou, China). The GRK2 shRNA and overexpression plasmid were transfected to FLS using Lipofectamine 3000 (Invitrogen, CA, USA) according to manufacturer's instructions.

### 2.10. Docking of MDP to the G Protein-Coupled Receptor Kinase 2 Structural Model

Docking simulation of MDP with GRK2 protein (PDB ID: 3KRW, human GRK2 in complex with Gbetgamma subunits and balanol) was carried out using the program Discovery Studio 2.1 (DS 2.1; Accelrys Software Inc.). The active sites and sphere of 10 A° were defined according to the reported important amino acid residues of GRK2 that are generated around the active site pocket of the BSAI model using C-DOCKER, a molecular dynamics (MD) simulated-annealing-based algorithm module from DS 2.1. As previously described [32], the structure of protein and substrate was subjected to energy minimization using chemistry at Harvard macromolecular mechanics force field as implemented in DS 2.1. A full potential final minimization was then conducted to refine the substrate poses. Based on C-DOCKER, energy docked conformation of the substrate was retrieved for postdocking analysis.

### 2.11. Imaging Flow Cytometry to Detect the Nuclear Expression of HIF-1*α*

The flow cytometry was performed following the earlier published standard protocol [33]. Cells were separated into different tubes and incubated with Cytofix and Cytoperm for simultaneous fixation and permeabilization. Next, cells were stained with a HIF-1*α* primary antibody according to manufacturer's directions and then sequentially incubated with the corresponding fluorescent secondary antibody and 2-(4-Amidinophenyl)-6-indolecarbamidine (DAPI). The images were detected by imaging flow cytometer (Amnis Imagestream Mark II), and positive images were analyzed by ideas 6.2 analysis.

### 2.12. Detection of the mRNA Expression Levels of NLRP3, ASC, and Caspase-1 by Quantitative PCR (qPCR)

Total RNA from the cells was extracted using TRIzol (Biomed, Beijing, China) reagent according to manufacturer's manual. Complementary DNA (cDNA) was synthesized using an oligo (dT) primer and PrimeScript™ RT Reagent Kit (Takara, Shiga, Japan). qPCR was performed to amplify the cDNA using the SYBR Premix Ex Tag Kit (Takara, Shiga, Japan) on an ABI 7500 Sequencing Detection System (Applied Biosystems, Foster City, CA, USA). Primers were designed and synthesized by Shanghai General Bio Service Company as per the gene sequences available in the GenBank, together with Oligo v6.6. Sequences for primers were as follows: NLRP3 forward 5′-CTCACCTCACACTCCTGCTG-3′, reverse 5′-AGAACCTCACAGAGCGTCAC-3′; Caspase-1 forward 5′-GACCGAGTGGTTCCCTCAAG-3′, reverse 5′-GACGTG TACGAGTGGGTGTT-3′. ASC forward 5′-GACAGTACCAGGCAG TTCGT-3′, reverse 5′-AGTAGGGCTGTGTTTGCCTC-3′. *β*-Actin, forward 5′-GGAGATTACTGCCCTGGCTCCTAGC-3′, reverse 5′-GGCCGGACTCATCGTACTCCTGCT-3′. The mRNA level of individual genes was normalized to *β*-actin and calculated by the 2^−ΔΔCt^ data analysis method.

### 2.13. Determination of Intracellular Reactive Oxygen Species

FLS were transferred after different treatments to different EP tubes. Next, cells were stained using the ROS Assay Kit (S0033S) according to the instructions. As previously described [33], the ROS content was estimated with a flow cytometer (FC500, Beckman); the results were analyzed using CytExpert (Beckman), and calculated by mean fluorescence intensity.

### 2.14. ROS Relative Quantification

An equal number of cells were planted in each well of the 96-well plate. The old medium was sucked up, and the cells were washed with PBS once. Under dark conditions, 100 *μ*L probe working solution was added to each well, and the cells incubated for 20 min at 37°C. Discarding the probe incubation solution, wash cells gently with PBS once. Then, add 100 *μ*L medium to each well. The fluorescence intensity was detected, and the data was saved. Then, the number of cells per well was estimated by CCK-8 method. And the ratio of fluorescence intensity to total cell number is the relative ROS level.

### 2.15. Fibroblast-Like Synoviocytes Proliferation Assay

FLS were counted and seeded in 96-well plates at a density of 1 × 10^4^ cells/well and incubated in DMEM at 37°C for 24 h. The cells were then exposed to hypoxia conditions for 24 h and treated with or without MDP for 16 h. After treatment, the cells were washed with PBS and incubated with DAPI dihydrochloride for 5 min. Finally, the number of cells per well was calculated by high-content analysis.

### 2.16. Statistical Analysis

The results were presented as the mean ± standard deviation of at least three separate experiments performed in triplicate. SPSS v16.0 software was used to analyze differences among groups by performing a one-way analysis of variance followed by Bonferroni postcomparison test (>two groups) or two-sample *t*-test (two groups) with significant differences at *p* < 0.05.

## 3. Result

### 3.1. Fibroblast-Like Synoviocytes pyroptosis under Hypoxia

**Figure 1:**
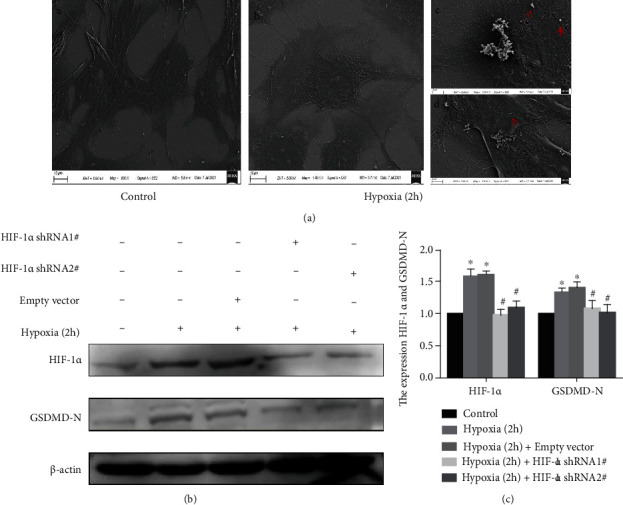
Hypoxia-mediated induction of pyroptosis in fibroblast-like synoviocytes (FLS). (a) Representative scanning electron microscope images of FLS pyroptosis. a, b FLS cultured under normoxia and hypoxia, respectively (1000x). c, d FLS cultured under hypoxia for 2 h (3000x). A HIF-1*α* short hairpin (shHIF-1*α*) construct was transfected into FLS, and hypoxic condition was imposed for 2 h. (b, c) Immunoblot analysis and quantification of hypoxia-inducible factor (HIF)-1*α* and N-terminal domain of human gasdermin D (GSDMD-N). Data are presented as mean ± standard deviation. *n* = 3. ^∗^*p* < 0.05 compared with the control group and ^#^*p* < 0.05 compared with the empty vector group.

To verify that hypoxia can induce FLS pyroptosis, we compared the morphological differences of FLS cultured under normoxia and hypoxia by electron microscopy. FLS exposed to a hypoxic environment exhibited a large number of membrane pores. Interestingly, under higher magnification, the cell contents overflowing from the membrane pores were also observed (Figure 1(a)). Next, Western blot analysis showed increased expression of GSDMD-N under hypoxia in FLS (Figures 1(b) and 1(c)). Meanwhile, the HIF-1*α* shRNA group showed a significant decrease in GSDMD-N compared with the negative control group (*p* < 0.05) (Figures 1(b) and 1(c)).

### 3.2. Monomeric Derivatives of Paeoniflorin-Mediated Reduction of the Hypoxia-Induced Pyroptosis of FLS

**Figure 2:**
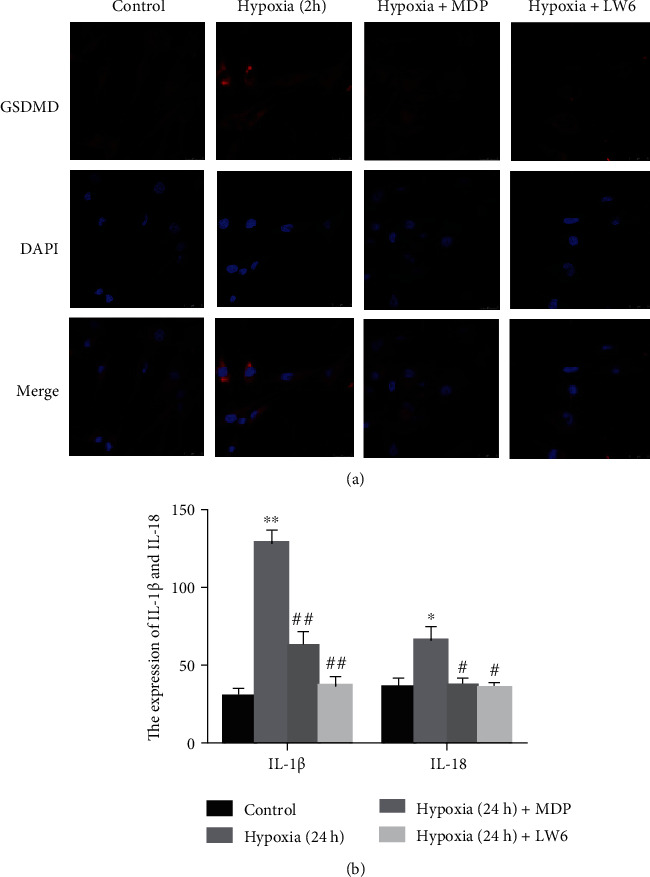
Monomeric derivatives of paeoniflorin (MDP) reduces hypoxia-induced fibroblast-like synoviocytes (FLS) pyroptosis. (a) Representative images of immunofluorescence of N-terminal domain of human gasdermin D (GSDMD-N) in FLS. (b) Enzyme-linked immunosorbent assay and quantification of interleukin- (IL-) 1*β* and IL-18 (*n* = 5). Data are expressed as mean + standard deviation. ^∗^*p* < 0.05, ^∗∗^*p* < 0.01 vs. the control group; ^#^*p* < 0.05, ^##^*p* < 0.01 vs. the hypoxia group.

Immunofluorescence image analysis showed that the level of GSDMD-N in FLS increased under hypoxia and decreased after MDP treatment (Figure 2(a)). Meanwhile, examination of the FLS supernatant revealed an increase in cytokines levels of IL-1*β* and IL-18 under hypoxia but decreased significantly after MDP treatment of FLS under hypoxia (Figure 2(b)).

### 3.3. Increased Expressions of Hypoxia-Inducible Factor-1*α* and Gasdermin D and the Inhibition of Monomeric Derivatives of Paeoniflorin in Fibroblast-Like Synoviocytes in Patients with Rheumatoid Arthritis and Animal Models of Arthritis

**Figure 3:**
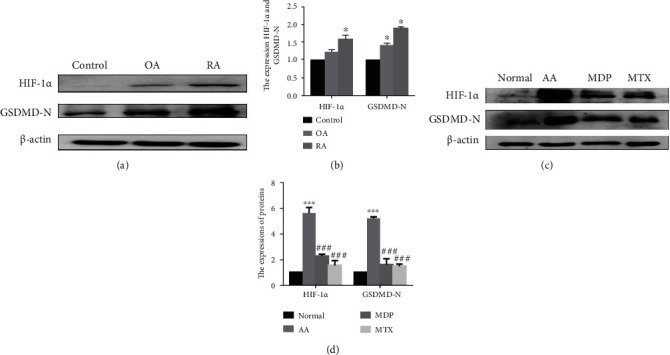
The levels of hypoxia-inducible factor (HIF)-1*α* and N-terminal domain of human gasdermin D (GSDMD-N) increase in fibroblast-like synoviocytes (FLS) of RA patients and adjuvant-induced arthritis (AA) rats. (a, b) Immunoblot analysis and quantification of HIF-1*α* and GSDMD-N in the FLS of RA patients. (c, d) Immunoblot analysis of HIF-1*α* and GSDMD-N expression levels in FLS of normal, AA, AA+MDP, and AA+MTX rats. Data are presented as mean ± standard deviation. *n* = 3. ^∗^*p* < 0.05, ^∗∗∗^*p* < 0.001 compared with the control group and ^###^*p* < 0.001 compared with the AA group (MTX: methotrexate; MDP: monomeric derivatives of paeoniflorin).

Western blot analysis showed highly expressed HIF-1*α* and GSDMD-N in the FLS of RA patients (Figures 3(a) and 3(b)). Higher expressions of HIF-1*α* and GSDMD-N were also observed in the FLS of adjuvant arthritis (AA) samples (Figures 3(c) and 3(d)) that were interestingly restored by MDP (Figures 3(e) and 3(f)).

### 3.4. Induction of FLS pyroptosis by Hypoxia through the Upregulated Expression of NLRP3 Inflammasome and the Role of Monomeric Derivatives of Paeoniflorin

**Figure 4:**
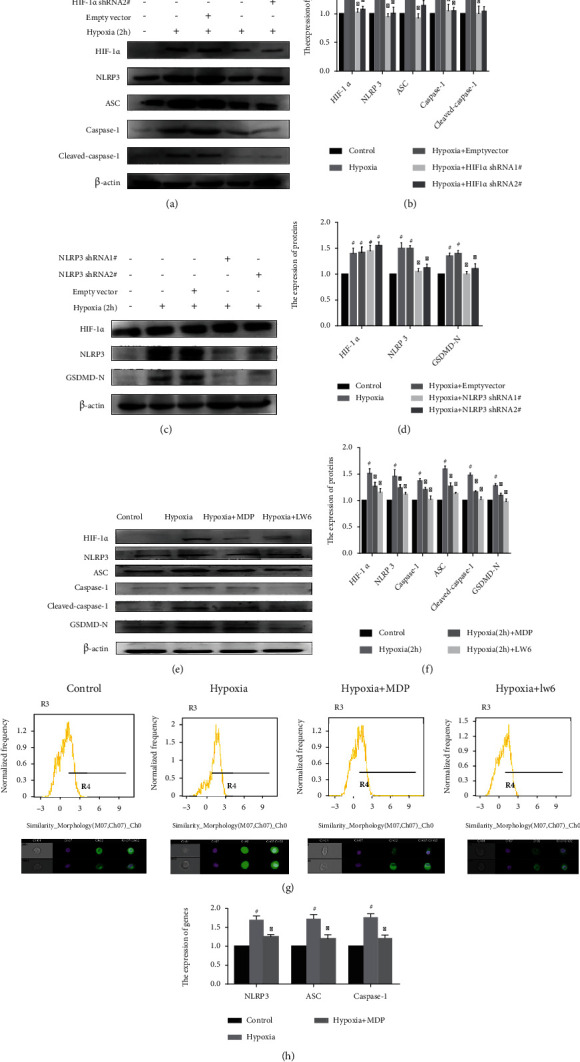
Hypoxia induces fibroblast-like synoviocytes (FLS) pyroptosis by upregulating the expression of nucleotide-binding oligomerization segment-like receptor family 3 (NLRP3) inflammasome in FLS and the effect of monomeric derivatives of paeoniflorin (MDP). (a, b) shHIF-1*α* was transfected into FLS and exposed to hypoxia for 2 h. The expression of hypoxia-inducible factor (HIF)-1*α*, NLRP3, speck-like protein containing CARD (ASC), caspase-1, and cleaved-caspase-1 in FLS was detected by Western blot (*n* = 3). (c, d) NLRP3 short hairpin (shNLRP3) construct was transfected into FLS and exposed to hypoxia for 2 h. The expression of HIF-1*α*, NLRP3, and N-terminal domain of human gasdermin D (GSDMD-N) in FLS after exposure to hypoxia for 2 h was detected by Western blot (*n* = 3). (e, f) FLS were treated with or without MDP and exposed to hypoxia for 2 h. The expression of HIF-1*α*, NLRP3, ASC, caspase-1, cleaved-caspase-1, and GSDMD-N in FLS was detected by Western blot (*n* = 3). Data are expressed as mean ± SD. ^#^*p* < 0.05, ^##^*p* < 0.01 vs. the control group; ^∗^*p* < 0.05, ^∗∗^*p* < 0.01 vs. the hypoxia group. (g) Analysis of expression levels of HIF-1*α* in the nucleus. (h) Quantitative PCR analysis and quantification of the mRNA levels of NLRP3, ASC, and caspase-1. Data are expressed as mean SD. ^#^*p* < 0.05 vs. the control group; ^∗^*p* < 0.05 vs. the hypoxia group.

To explore the mechanism of hypoxia-induced FLS pyroptosis, we tested the levels of NLRP3 inflammasome and cleaved-caspase-1 which were closely related to pyroptosis. Western blot showed a significant increase in the contents of NLRP3 inflammasome and cleaved-caspase-1 in FLS under hypoxia. Further, after HIF-1*α* knockdown, the levels of NLRP3 inflammasome and cleaved-caspase-1 also reduced (Figures 4(a) and 4(b)). The expression of GSDMD-N also decreased in the absence of NLRP3 (Figures 4(c) and 4(d)). Treatment with MDP led to a decrease in the levels of NLRP3 inflammasome and cleaved-caspase-1 (Figures 4(e) and 4(f)).

Then, to confirm that the transcription of NLRP3 inflammasome is directly regulated by HIF-1*α*, the proportion of HIF-1*α* in the nucleus and mRNA expression level of NLRP3 inflammasome were determined. The expression of HIF-1*α* in the nucleus increased under hypoxic conditions, as shown by flow cytometry imaging (Figure 4(g)). Meanwhile, the mRNA levels of NLRP3 inflammasome increased significantly in FLS under hypoxia (Figure 4(h)). However, MDP could suppress this change (Figures 4(g) and 4(h)).

### 3.5. Hypoxia Increases HIF-1*α* Expression by Upregulating G Protein-Coupled Receptor Kinase 2 Expression in Fibroblast-Like Synoviocytes

**Figure 5:**
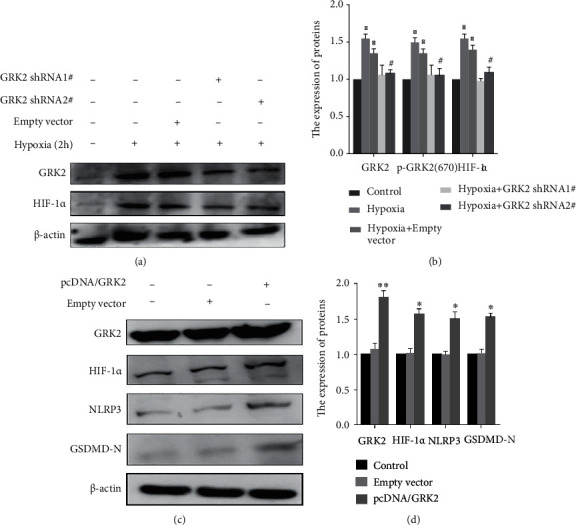
G protein-coupled receptor kinase 2 (GRK2) promotes the expression of hypoxia-inducible factor (HIF)-1*α*. (a, b) shGRK2 was transfected into fibroblast-like synoviocytes (FLS) and exposed to hypoxia for 2 h. Immunoblot analysis and quantification of GRK2 and HIF-1*α*. (c, d) GRK2 overexpression plasmid was transfected into FLS. Immunoblot analysis and quantification of GRK2, HIF-1*α*, nucleotide-binding oligomerization segment-like receptor family 3 (NLRP3), and N-terminal domain of human gasdermin D (GSDMD-N). The values are shown as the mean ± standard deviation (*n* = 3). ^∗^*p* < 0.05, ^∗∗^*p* < 0.01 compared with the control group and ^#^*p* < 0.05 compared with the hypoxia group or the NC group.

To further clarify the relationship between GRK2 and HIF-1*α*, GRK2-knockdown and GRK2-overexpressing cells were examined Unsurprisingly, HIF-1*α* expression decreased in absence of GRK2 (Figures 5(e) and 5(f)) and increased in the presence of excess GRK2 (Figures 5(g) and 5(h)).

### 3.6. MDP Reduces HIF-1*α* Expression through Targeted Inhibition of G Protein-Coupled Receptor Kinase 2S670 Phosphorylation in Fibroblast-Like Synoviocytes

**Figure 6:**
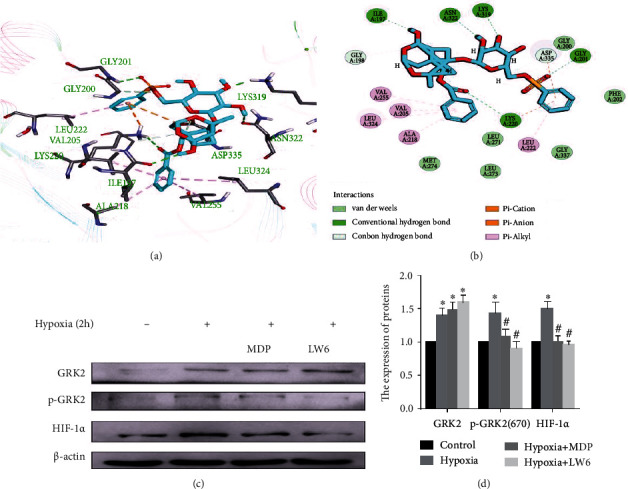
Monomeric derivative of paeoniflorin (MDP) inhibits the expression of hypoxia-inducible factor (HIF)-1*α* through targeted inhibition of GRK2 S670 phosphorylation. (a) Molecular docking modeling of the compound MDP and G protein-coupled receptor kinase 2 (GRK2), the small molecule, and the critical interaction of 3KRW (human GRK2 in complex with Gbetgamma subunits and balanol) are represented by sticks. (b) A schematic representation of the binding mode of MDP in the GRK2 binding site of 3KRW. (c, d) FLS were treated with or without MDP and exposed to hypoxia for 2 h. Immunoblot analysis and quantification of GRK2, p-GRK2 (S670), and HIF-1*α*. The values are shown as the mean ± standard error of the mean (*n* = 3). ^∗^*p* < 0.05 compared with the control group and ^#^*p* < 0.05 compared with the hypoxia group.

The molecular docking assay showed the formation of hydrogen bonds between I197, K319, K220, N322, and G201 in the kinase domain of GRK2 and MDP (Figures 6(a) and 6(b)). Further research showed that MDP inhibited the phosphorylation of GRK2 S670, rather than reducing the expression level of GRK2 (Figures 6(c) and 6(d)).

### 3.7. The Increase in G Protein-Coupled Receptor Kinase 2 Level under Hypoxia Is due to the Increase in Reactive Oxygen Species Content in Fibroblast-Like Synoviocytes

**Figure 7:**
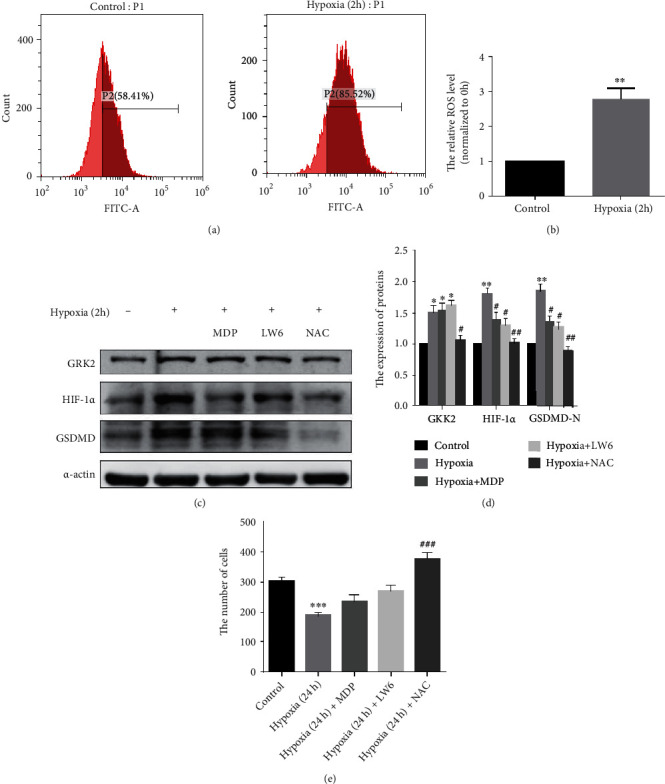
Excessive reactive oxygen species (ROS) upregulates the expression level of G protein-coupled receptor kinase 2 (GRK2) under hypoxia in fibroblast-like synoviocytes (FLS). (a, b) The analysis and quantification of ROS in FLS. (c, d) FLS were pretreated with N-acetyl-l-cysteine (NAC) before exposure to hypoxia. Immunoblot analysis and quantification of GRK2, hypoxia-inducible factor (HIF)-1*α*, and N-terminal domain of human gasdermin D (GSDMD-N) in FLS. (e) Cell proliferation analysis.

To explore the cause for the increase in GRK2 under hypoxic conditions, the ROS content was assessed. Flow cytometry data and the quantitative results of enzyme reader showed that ROS levels increased significantly after 2 h of hypoxia in FLS (Figures 7(a) and 7(b)). Besides, the levels of GRK2, HIF-1*α*, and GSDMD-N reduced significantly in FLS after NAC (a ROS scavenger) treatment (Figures 7(c) and 7(d)). In addition, the high-content analysis showed a decrease in the number of cells after FLS were exposed to a hypoxic environment for 24 h (Figure 7(e)).

## 4. Discussion

We herein present evidence for hypoxia aggravated synovitis by inducing FLS pyroptosis in RA. Our results showed that removal of ROS, HIF-1*α*, GRK2, or NLRP3 knockdown significantly attenuated hypoxia-induced FLS pyroptosis. Moreover, ROS promoted HIF-1*α* synthesis by upregulating GRK2 expression.

The well-known, most obvious characteristic of RA is synovitis [34]. Hypoxia in synovial tissues is one of the important pathological features of synovitis [35]. HIF-1*α* is especially prominent in RA pathogenesis because it contributes to almost all aspects of RA-related pathologies, including synovial inflammation, damage of cartilage and bone, and angiogenesis [36]. Therefore, we performed this study demonstrating that the FLS pyroptosis, a proinflammatory mechanism, was triggered by HIF-1*α*. Indeed, several studies have shown that HIF-1*α* is an important cause of pyroptosis [37]. For example, microglia pyroptosis is thought to be caused by elevated HIF-1*α* in stroke [38]. Cell pyroptosis is involved in the occurrence and development of RA, and there may be articular cartilage cell pyroptosis during the process [39]. Interestingly, the serum from RA patients was found to induce GSDMD-dependent pyroptosis in monocytes, and this ability was associated with disease activity [40]. Inconsistent with these findings, the current study demonstrated that the synovial hypoxic microenvironment aggravates synovitis by inducing FLS pyroptosis in RA.

Pyroptosis is mainly mediated by the NLRP3 inflammasome [41–43]. NLRP3 gene polymorphism is associated with susceptibility to RA [44]. Kolly and colleagues indicated that endothelial and inflammatory cells in RA synovium express all components needed for inflammasome activation [45, 46]. The treatment of RA patients with MCC950, a targeted inhibitor of NLRP3, resulted in significantly less severe joint inflammation and bone destruction and reduced production of IL-1*β* [44]. These studies show the vital role of NLRP3 inflammasome in the pathological process of RA. Cosin-Roger and his colleagues discovered one binding site for HIF-1*α* at −150 in the NLRP3 promoter through chromatin immunoprecipitation [47]; HIF-1*α* regulates inflammatory responses through the NLRP3 inflammasome, thus influencing both apoptotic and pyroptotic cell death after stroke [48]. In this study, we found an essential role of NLRP3 in HIF-1*α*-induced FLS pyroptosis and the regulation of the NLRP3 inflammasome mRNA level by HIF-1*α*. Accordingly, we propose that the HIF-1*α*-induced FLS pyroptosis was achieved by activating the NLRP3 inflammasome in RA.

The finding that MDP inhibits macrophage pyroptosis both in vivo and in vitro seems to give us a strategy to treat RA, although the mechanism is currently not known. One possible mechanism is through the inhibition of GRK2 phosphorylation, given that the phosphorylation of GRK2 S670 positively regulates HIF-1*α* [15]. Interestingly, the phosphorylation of GRK2 at S670 is essential for the translocation of GRK2 to the mitochondria of cardiomyocytes postischemia reperfusion injury in vitro, and that this localization promotes cell death [49]. From this perspective, MDP likely exerts a protective effect by inhibiting phosphorylation of GRK2 S670 in FLS. It is also noteworthy that the phosphorylation of GRK2 S670 facilitates mouse double minute 2 (an E3 ubiquitin ligase to the receptor complex)-mediated GRK2 degradation [50, 51]. Indeed, the expression levels of GRK2 increased slightly in FLS after MDP treatment under hypoxia, which indicates that MDP may only inhibit GRK2 phosphorylation rather than its expression. However, Chang et al. demonstrated that Pae suppresses the proliferation of FLS and decreases GRK2 expression in FLS in vitro, indicating that MDP may have a role in reducing GRK2 expression [52, 53]. The expression of GRK2 was also inhibited by MDP in AA synovial tissue.

To explain this phenomenon, our follow-up experiments revealed that the increase in GRK2 levels under hypoxic conditions was due to the large accumulation of ROS. Increased levels of ROS at the site of inflammation were found to cause cell damage and the progression of inflammatory disease [54, 55]. Under hypoxia, the level of ROS increased significantly in FLS due to a wide spectrum of alterations in mitochondrial structure, dynamics, and genome stability [56]. The accumulation of ROS activates a negative feedback loop in response to oxidative stress [57, 58]. Indeed, the accumulation of the ROS lasts only for a short period under hypoxia, indicating the activation of ROS clearance mechanism in FLS. Recent studies have discovered many ROS clearance mechanisms, such as mitophagy, the activation of superoxide dismutase, and glutathione peroxidase [57, 59]. Interestingly, the highly expressed HIF-1*α* can activate mitophagy by increasing BNIP3 transcription [59]. Therefore, in vitro inhibition of HIF-1*α* expression may cause a transient increase in ROS and GRK2 levels in FLS under hypoxic conditions.

In our experiments, MDP significantly inhibited HIF-1*α* expression, which in turn prevented NLRP3 inflammasome activation, resulting in reduced pyroptosis in FLS under hypoxia. Particularly, GSDMD silencing can significantly reduce both gene and protein levels of fibrogenic markers transforming growth factor-*β*, procollagen-lysine 2-oxoglutarate 5-dioxygenase2, collagen type I *α*1 chain, and tissue inhibitor of metalloproteinases 1 [37]. Therefore, MDP may have the ability to relieve synovial fibrosis. Pyroptosis can influence the proliferation, invasion, and metastasis of tumors, which are regulated by some noncoding RNAs and other molecules [60]. FLS of RA have tumor-like growth characteristics, further proving that hypoxia-induced FLS pyroptosis plays a vital role in the course of RA [61]. In summary, MDP, as a natural drug-derived HIF-1*α* inhibitor, has great potential for treating RA. MDP may be a multitarget HIF-1*α* inhibitor, because in the process of research, surprisingly, we found that MDP could induce VHL production.

**Figure 8:**
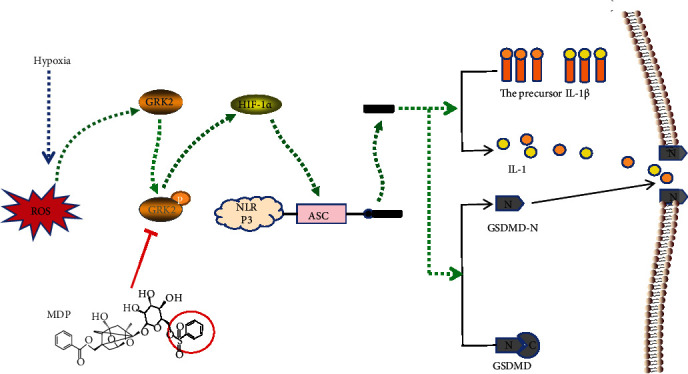
Reactive oxygen species/G protein-coupled receptor kinase 2/hypoxia-inducible factor-1*α*/nucleotide-binding oligomerization segment-like receptor family 3- (ROS/GRK2/HIF-1*α*/NLRP3-) mediated pyroptosis in fibroblast-like synoviocytes (FLS) promotes synovitis under hypoxia and the regulation by monomeric derivatives of paeoniflorin (MDP). The level of ROS in FLS rises sharply under hypoxia. Then, excessive ROS promotes GRK2 expression and increases the levels of phosphorylated GRK2 S670, which then increases HIF-1*α* synthesis. This elevated HIF-1*α* is transferred to the nucleus to initiate the transcription of the NLRP3 inflammasome. The abnormally high level of NLRP3 inflammasome is activated under hypoxia to form activated cleaved-caspase-1, which, in turn, shears gasdermin D (GSDMD), interleukin- (IL-) 1*β*, and IL-18 to induce FLS pyroptosis. Monomeric derivative of paeoniflorin (MDP) inhibits the phosphorylation of GRK2 S670 to reduce FLS pyroptosis and relieve synovitis.

In conclusion, the data presented here clearly demonstrated that hypoxia induces FLS pyroptosis by activating the ROS/GRK2/HIF-1*α*/NLRP3 pathway and MDP could reduce hypoxia-induced FLS pyroptosis by inhibiting the GRK2/HIF-1*α* axis (Figure 8). In this regard, MDP holds great potential to be clinically translated as a therapeutic agent for RA management.

## Figures and Tables

**Table 1:** Composition of short hairpin RNAs.

HIF-1*α*1#	Sense	5′-AATCAAAAGCAGTGACGAA-3′
	Antisense	5′-TTCGTCACTGCTTTTGATT-3′
HIF-1*α*2#	Sense	5′-CTGATAACGTGAACAAATA-3′
	Antisense	5′-TATTTGTTCACGTTATCAG-3′
NLRP3 1#	Sense	5′-CCUGUCUUUGCCGUAGAUUACCGUAAG-3′
	Antisense	5′-CUUACGGUAAUCUACGGCAAAGACAGG-3′
NLRP3 2#	Sense	5′-GUGGACCUCAAGAAAUUUATT-3′
	Antisense	5′-UAAAUUUCUUGAGGUCCACTT-3′
GRK2 1#	Sense	5′-CCAUGAAGUGUCUGGACAATT-3′
	Antisense	5′-UUGUCCAGACACUUCAUGGTT-3′
GRK2 2#	Sense	5′-GCAGGUACCUCCAGAUCUC-3′
	Antisense	5′-GAGAUCUGGAGGUACCUGC-3′

## Data Availability

The data used to support the findings of this study are available from the corresponding author upon request.

## Supplementary Materials

Supplementary Materials Extended Data Figure 1: monomeric derivatives of paeoniflorin (MDP) can inhibit fibroblast-like synoviocytes pyroptosis induced by Co^2+^. Extended Data Figure 2: the levels of G protein-coupled receptor kinase 2 (GRK2), hypoxia-induced factor-1*α*, and reactive oxygen in fibroblast-like synoviocytes were significantly increased in FLS after 2 h of hypoxia. Extended Data Figure 3: monomeric derivatives of paeoniflorin- (MDP-) mediated inhibition of GRK2 expression and induced Von Hippel-Lindau (VHL) production in fibroblast-like synoviocytes of adjuvant-induced arthritis (AA) rats.
