# Supplementary material for: The ROS/GRK2/HIF-1α/NLRP3 Pathway Mediates Pyroptosis of Fibroblast-Like Synoviocytes and the Regulation of Monomer Derivatives of Paeoniflorin
Source: Oxid Med Cell Longev. 2022 Jan 29;2022:4566851. doi: 10.1155/2022/4566851 (PMC8817856; doi:10.1155/2022/4566851)
Supplement: Supplementary Materials — Extended Data Figure 1: monomeric derivatives of paeoniflorin (MDP) can inhibit fibroblast-like synoviocytes pyroptosis induced by Co2+. Extended Data Figure 2: the levels of G protein-coupled receptor kinase 2 (GRK2), hypoxia-induced factor-1α, and reactive oxygen in fibroblast-like synoviocytes were significantly increased in FLS after 2 h of hypoxia. Extended Data Figure 3: monomeric derivatives of paeoniflorin- (MDP-) mediated inhibition of GRK2 expression and induced Von Hippel-Lindau (VHL) production in fibroblast-like synoviocytes of adjuvant-induced arthritis (AA) rats. [file 4566851.f1.pdf]

Extended Data Fig. 1 MDP can inhibit FLS pyrolysis induced by Co^{2+} .

(A, B) FLS were treated with Co^{2+} for 24h and different doses of MDP for 16h. Immunoblot analysis and quantification of HIF-1α and GSDMD-N. (C, D) FLS were treated with Co^{2+} for 24h and MDP (10^{-6} mol/L) for 16h.

Extended Data Fig. 2 After 2 hours of hypoxia, the levels of GRK2, HIF-1α and ROS were significantly increased in FLS.

(A, B)Immunoblot analysis and quantification of HIF-1α, NLRP3, ASC, Caspase-1, and cleaved-Caspase-1. FLS was exposed to hypoxia for 0, 1, 2, 3, 4, 6, 24, 48h. Immunoblot analysis and quantification of GRK2, HIF-1α. (C) The images of ROS fluorescent.

Extended Data Fig. 3 MDP could inhibit the expression of GRK2 in FLS of AA rats and induce VHL production

(A, B) Immunoblot analysis and quantification of HIF-1α and GSDMD-N in the FLS of different groups of rats. Data were presented as mean ± SD. n = 3. ***$p < 0.001$ compared with control group and $^{###}p < 0.001$ compared with AA group. (C, D) The expression level of VHL treated with or without MDP. Data were presented as mean ± SD. n = 3. *$p < 0.05$ compared with control group.

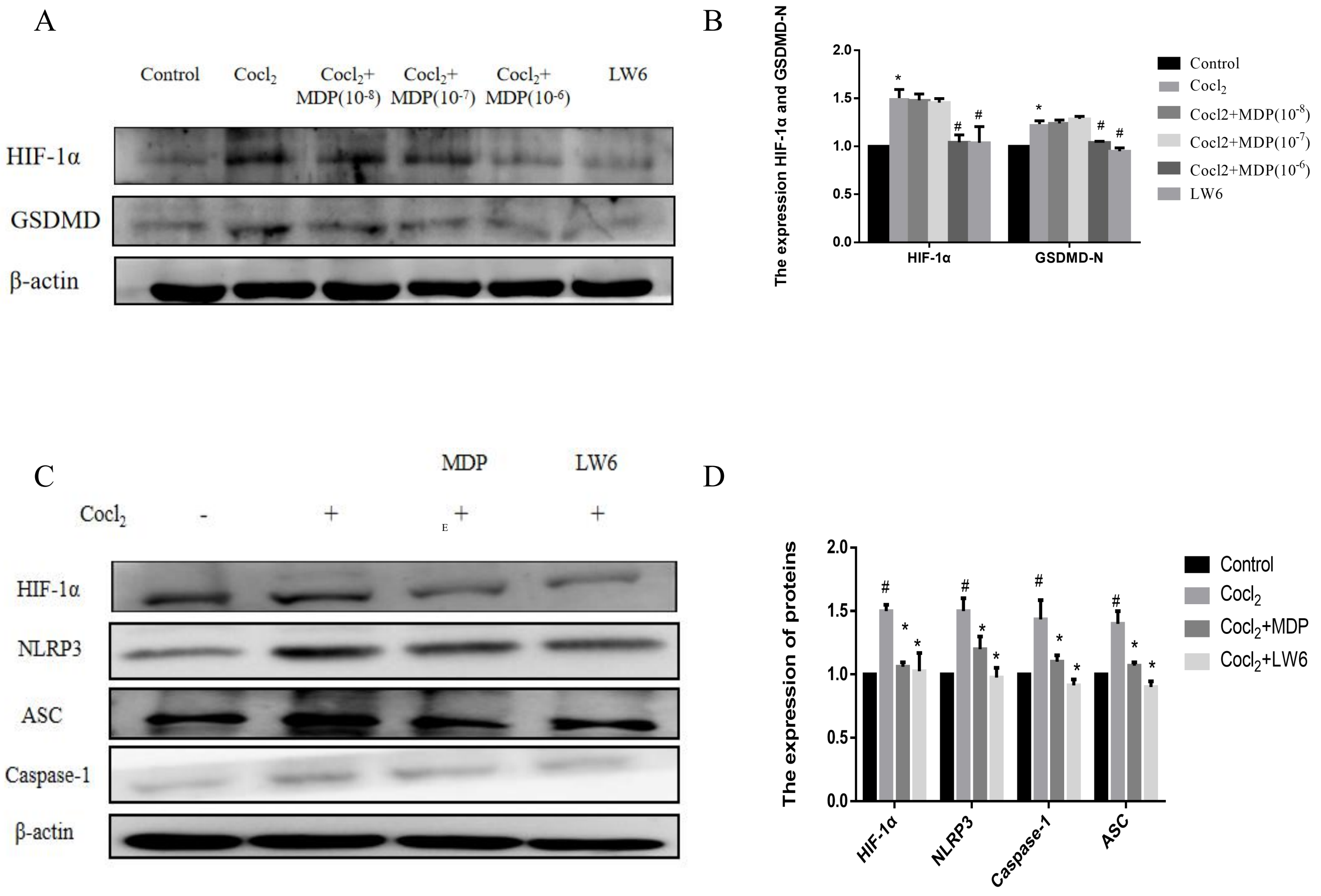

Extended Data Fig. 1

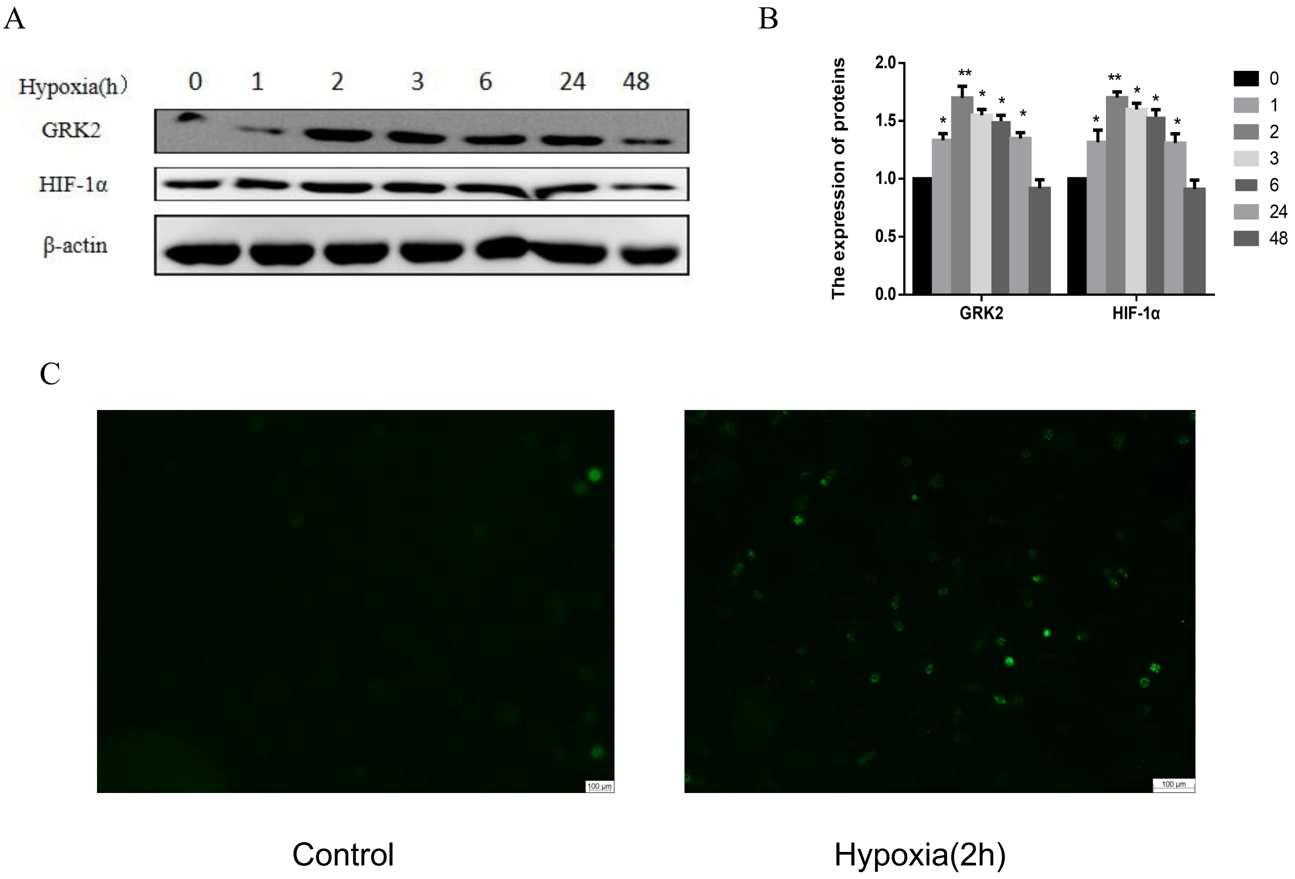

Extended Data Fig. 2

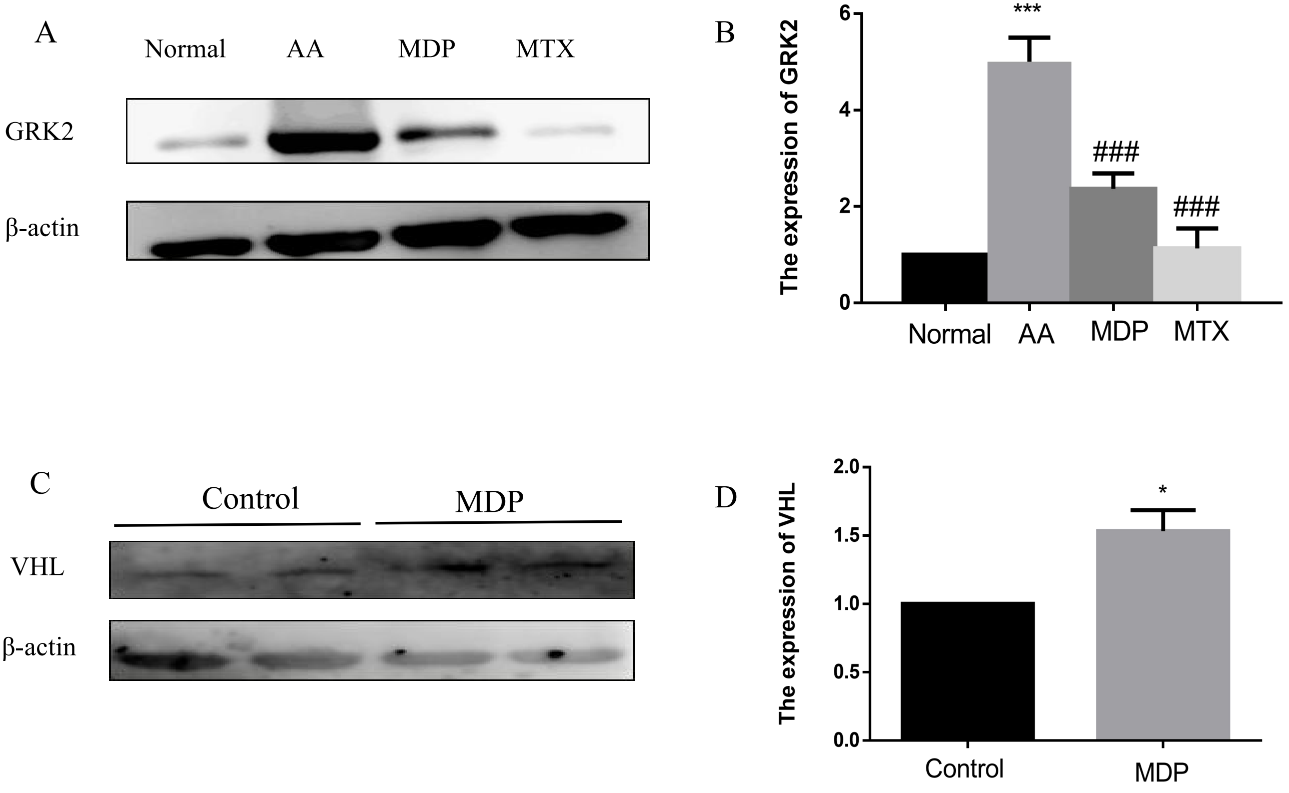

Extended Data Fig. 3
